# Effect of mesenchymal stem cells on cytochrome-*c* release and inflammation in colon cancer induced by 1,2-dimethylhydrazine in Wistar albino rats

**DOI:** 10.1042/BSR20204356

**Published:** 2021-03-02

**Authors:** Afrah F. Alkhuriji, Seham G. Alsaiari, Suliman Y. Alomar, Alaa A. Alnafjan, Hussah Alobaid, Manal F. El-Khadragy

**Affiliations:** 1 Department of Zoology, College of Science, King Saud University, Riyadh 11495, Saudi Arabia; 2 Department of Biology, Al-Nairiyah University College, University of Hafr Al-Batin, Hafr Al-Batin 31991, Saudi Arabia; 3 Doping Research Chair, Department of Zoology, College of Science, King Saud University, 11 Riyadh 11495, Saudi Arabia; 4 Biology Department, Faculty of Science, Princess Nourah Bint Abdulrahman University, Riyadh 11671, Saudi Arabia; 5 Department of Zoology and Entomology, Faculty of Science, Helwan University, Cairo 11795, Egypt

**Keywords:** 1,2-dimethylhydrazine, colon cancer, cytochrome-c, mesenchymal stem cells

## Abstract

Colon cancer is one of the most common causes of deaths by cancer worldwide. Stem cells have immunosuppressive properties that promote tumor targeting and circumvent obstacles currently in gene therapy. Bone marrow stem cells are believed to have anticancer potential. The transplantation of mesenchymal stem cells (MSCs), a type of bone marrow stem cells, has been considered a potential therapy for patients with solid tumors due to their capability to enhance the immune response; MSC transplantation has received renewed interest in recent years. The present study aimed to evaluate the antiapoptotic effects of the MSCs on 1,2-dimethylhydrazine (DMH)-induced inflammation in the rat model of colorectal cancer. The rats were randomly allocated into four groups: control, treated with MSCs, induced by DMH, and induced by DMH and treated with MSCs. The MSCs were intra-rectally injected, and DMH was subcutaneously injected at 20 mg/kg body weight once a week for 15 weeks. The administration of MSCs into rats starting from day 0 of the DMH injection was found to enhance the histopathological picture. The MSC treatment resulted in fewer inflammatory cells than in the DMH group. Therefore, our findings suggest that BMCs have antitumor effects by modulating the cellular redox status and down-regulating the pro-inflammatory genes. Thus, BMCs may provide therapeutic value for colon cancer treatment.

## Introduction

Colorectal cancer is one of the most common cancers and a significant cause of morbidity and mortality worldwide. Colon cancer ranks first in males and third in females in the Kingdom of Saudi Arabia (KSA) among all the cancer types. According to the Saudi Cancer Registry report in 2019, the incidence of all cancers, including colorectal cancer, in Saudi Arabia has gradually increased [1,2].

The potential risk factors for colorectal cancer include behavioral factors, such as lifestyle, a diet of low fruit and vegetable intake, and biological factors, such as genetic mutations and hormonal dysfunction [4]. Colorectal carcinogenesis is a multistep process induced by the genetic modifications of various tumor suppressor genes and oncogenes that convert normal colonic epithelia into metastatic carcinoma [3]. Also, colon cancer is frequently a pathological consequence of persistent oxidative stress that causes DNA damage, mutations in cancer-related genes, and a cycle of cell death, mutations, and regeneration involving the overproduction of reactive oxygen species (ROS) and reactive nitrogen species (RNS).

Currently, colon cancer is treated with surgery, chemotherapy, radiation therapy, immunotherapy, and nutritional-supplement therapy. However, the success rate of individual colon cancer therapy remains low due to secondary complications. Tumor cells quickly become resistant to anticancer drugs due to their genomic instability; hence, the development of novel drugs with a new mechanism and improved therapeutic efficacy is urgently needed [20,21].

Stem cell therapy is promising for treating degenerative or inherited diseases because they undergo self-renewal and differentiate into multiple cell types, such as osteoblasts, chondrocytes, adipocytes, and neurons [14,15]. Compared with embryonic stem cells, bone marrow-derived stem cells are the more promising option for treatment because it is believed to have anticancer potential and is preferred for its ability to stimulate the immune system and migrate to the site of tumors [5,6].

BMCs are an excellent source of mesenchymal stromal cells (MSCs) and hematopoietic stem cells (HSCs). BMC treatment involves allotransplantation, which is the transplantation of an organ or tissue from one individual to another of the same species with different genotypes. It can activate the immune response, affect the immune cell populations, and induce a robust allogeneic response, thus preventing tumor development [13].

While HSC transplantation has been employed in other cancers, such as breast cancer [16], MSCs have garnered much interest owing to their capacity to improve the immune response to cancer [17–19]. MSCs transplantation is applicable for treating patients with solid tumors. Thus, we hypothesized that the colorectal delivery of MSCs would be more effective than systemic administration in alleviating inflammation, oxidative stress, and apoptosis in colon cancer.

In the present study, a colon cancer model in rats was established using 1,2-dimethylhydrazine (DMH), a carcinogen usually used in the rodent carcinogenesis model. DMH is a hydrazine in which one of its hydrogens attached to the nitrogen has been replaced by a methyl group; it is a carcinogenic agent as well as an alkylating agent. DMH affects various organs; it induces tumors, especially in the distal colon, and causes pathological features such as aberrant crypt foci [7–10]. DMH causes the significant loss of colonic cells by increase the proliferation of colonic epithelial cells and gene mutations [11,12]. In the present study, we studied the effect of MSC treatment on the histology in rats with induced colon cancer. Also, we examined the effect of MSCs on the level of cytokeratin, oxidative stress, apoptosis, and cell proliferation in rats with colon cancer.

## Materials and methods

### Bone marrow-MSC culture protocol

All the animal bone samples were processed within 30 min of the animal’s death to ensure the bone marrow- mesenchymal stem cells (BM-MSCs) collected were highly viable. The animal’s tibias, femurs, and humeri were cut at the joints, placed on to sterile gauze, carefully scrubbed to remove the residual soft tissues, and transferred to a 100-mm sterile culture dish with 10 ml of complete α-MEM medium on ice. In a biosafety cabinet, the bones were washed twice with PBS containing 1% PSN to flush away the blood cells and the residual soft tissues, and then transferred to a new 100-mm sterile culture dish with 10 ml of complete α-MEM medium. A bone was stabilized with forceps, and its two ends were excised just below the end of the marrow cavity using micro dissecting scissors. Next, a needle was inserted into the bone cavity to flush out the marrow slowly, and the bone cavity was washed until it the bones became pale. The dish containing the marrow was incubated at 37°C in a 5% CO_2_ incubator for 5 days. According to the analysis by phase-contrast microscopy, the initial spindle-shaped cells appeared on day 3, and the culture reached 70–90% confluence within 2 days. The cells were washed with PBS twice, digested with 2.5 ml of 0.25% trypsin for 2 min at 37°C, and neutralized with 7.5 ml of complete α-MEM medium. Washing the cells with PBS before digestion was important, as it removed the residual medium and cell secretion and loosened the adhesive of the MSCs to the dish. The bottom of the plate was flushed using a pipette to displace the cells. The cells were then transferred to a 15-ml Falcon tube (Becton Dickinson), centrifuged at 800×***g*** for 5 min, and re-suspended in a 25-cm^2^ cell culture flask (Thermo Scientific™). Cell passaging was performed every 4–6 days, split at the ratio of 1:3, and counted in the Neubauer’s chamber using Trypan Blue to evaluate cell viability.

The cells were then injected into the intra-colorectal pathway of a rat. The rat colon was lavaged with 2 ml of saline. One milliliter of BM-MSCs at 6 × 10^6^ cells/ml in 0.9% saline was inoculated intra-rectally by a cannula that was introduced through the anus [21,22].

### Colon cancer induction

The chemical DMH was thawed in a solution containing 1 mM ethylenediaminetetraacetic acid and 1 mM sodium bicarbonate pH 6.5. The animals received a subcutaneous injection of DMH at 20 mg/kg body weight in the groin every week for 15 weeks [6].

### Animals and experimental ethics protocol

Forty male Wistar albino rats (Animal House of King Saud University, Faculty of Science, Riyadh, Saudi Arabia), aged 10 weeks and weighing 180–220 g, were obtained. The rats were housed in wired polypropylene cages at 25 ± 2°C and 55–60% humidity under a 12-h light/dark cycle for 2 weeks. They were fed a standard laboratory diet with unlimited access to water.

A total of 40 rats (*n*=40) were utilized to investigate the effects of BM-MSCs on DMH-induced colon cancer. The animals were randomly assigned to four groups with 10 (*n*=10) rats per group. The rats received a weekly subcutaneous injection of saline at 0.2 ml/week (group 1, control) or DMH at 20 mg/kg (group 2, DMH), a weekly intra-rectal application of BM-MSCs 6 × 10^6^ cells in saline (group 3, MSC), or both the DMH at 20 mg/kg and 6 × 10^6^ cells MSCs in saline (group 4, DMH and MSCs). All the application regimens lasted for 15 weeks [22]. At the final week of administration, the animals were sacrificed by CO_2_ asphyxiation. Their colons were immediately excised, cleaned to remove irrelevant materials, and frozen at −80°C without formalin for future molecular and biochemical examinations.

All the experiments were performed in compliance with the requirements of the local animal ethics committee of the Faculty of Science, King Saud University. Animal Care at the Zoology Department, Faculty of Science, King Saud University (IRB number: KSU-SE-18-35).

### Histochemical and histological staining

The colon was placed in 10% neutral buffered formalin for 24 h, washed in running water, dehydrated in ethyl alcohol, cleared in xylene, mounted in molten Paraplast, cut into 4–5-μm sections, and stained with Eosin and Hematoxylin, Periodic acid–Schiff (PAS), or Masson’s trichrome. Finally, the samples were observed using a Nikon microscope (Eclipse E200-LED, Tokyo, Japan).

### Immunohistochemistry

The colon, already cut into 4–5-μm sections, were incubated with an anti-Cytokeratin 20 primary antibody (Lab Vision, NeoMarkers, U.S.A.) for 90 min. Subsequently, a secondary antibody was applied according to the immunoperoxidase method (Vectastain ABC Kit; Vector Laboratories, Burlingame, CA).

### Measurement of the mitochondrial levels of cytochrome-*c*

The mitochondrial cytochrome-*c* levels were quantified using an enzyme-linked immunosorbent assay (ELISA) kit following the manufacturer’s instructions (cat. no. ab59348; Abcam, U.S.A).

### Measurement of oxidative stress markers

The level of malondialdehyde in a colon homogenate was quantified as an indicator of lipid peroxidation (LPO) [23].

### Quantification of antioxidant enzymatic activity

Various enzymatic activities in the colon homogenates were measured using ELISA kit according to the manufacturer’s instructions. Superoxide dismutase (SOD) activity was evaluated depending on its capacity to inhibit the phenazine methosulfate (PMS)-mediated reduction in the nitroblue tetrazolium dye to diformazan, according to Nishikimi et al. [24]. Catalase (CAT) activity was evaluated based on the conversion of hydrogen peroxide (H_2_O_2_) into water and oxygen at 240 nm, as shown by Aebi [25].

### Measurement of colorectal apoptotic biomarkers

The levels of Bcl-2 and Bax protein were measured in colon tissue lysates using the ELISA kit according to the manufacturer’s instructions.

### Gene expression analysis

Total RNA was extracted from the frozen colon samples using TRIzol. Approximately 5 µg of total RNA was reverse-transcribed to cDNA and quantified for *Tnf, Tgfb, Nos2*, or *Il1b* using real-time polymerase chain reaction (RT-PCR) with the Power SYBR® Green Master Mix Kit and the corresponding primers sets (Table 1). The glyceraldehyde 3-phosphate dehydrogenase gene (*Gapdh*) was utilized as the reference housekeeping gene.

**Table 1:** Primer sequences of the genes analyzed in a real-time polymerase chain reaction

Name	Sense (5′–3′)	Antisense (5′–3′)
*Tnf* (Tumor necrosis factor α)	AGAACTCAGCGAGGACACCAA	GCTTGGTGGTTTGCTACGAC
*Tgfb* (Transforming growth factor β)	TAGGCTGACAGCTTTGCGAA	GAACAACCGGCCTCCAAAAC
*Nos2* (Inducible nitric oxide synthase)	GTTCCTCAGGCTTGGGTCTT	TGGGGGAACACAGTAATGGC
*Il1b* (Interleukin 1 β)	GACTTCACCATGGAACCCGT	GGAGACTGCCCATTCTCGAC
*Gapdh* (Glyceraldehyde 3-phosphate dehydrogenase gene)	GCATCTTCTTGTGCAGTGCC	GATGGTGATGGGTTTCCCGT

### Statistical analysis

The data were represented as means ± standard errors of the means (SEMs). The significance of the differences between the groups was determined by the one-way analysis of variance, followed by Duncan’s multiple range tests. A difference between the groups was considered statistically significant at *P*-values <0.05.

## Results

### Colon cancer induces histological changes in rats

**Figure 1:**
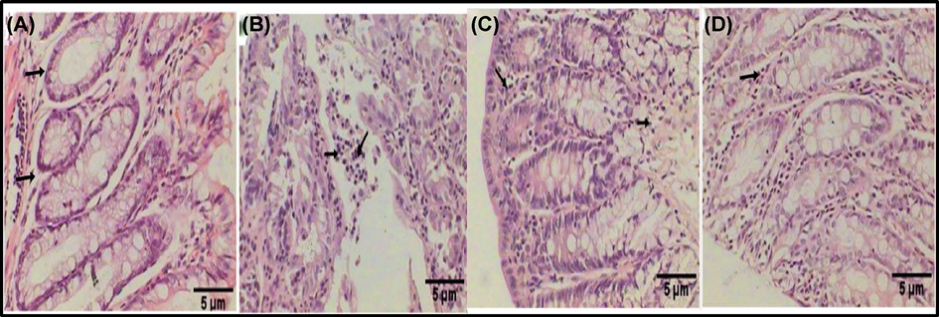
Histological changes of the colon section with Eosin and Hematoxylin Examination of the histological changes of the colon section of the control group exhibiting a normal histological structure of the glandular cells of the mucosal layer (**A**). Rat colon exhibiting tumor cells (anaplasia, dysplasia, and hyperchromasia) in the lumen (black arrows) after tumor induction by DMH (20 mg/kg) (**B**). A photomicrograph of the colon section of cancer-prompted rat treated with MSCs, exhibiting a clear enhancement in the lining epithelium of the mucosa, and a few inflammatory cells (black arrows) (**C**). A photomicrograph of a colon treated rat exhibiting a healthy structure (**D**) (HE & E: 400×).

The colon sections from the rats in the control group showed that the glandular cells of the mucosal layer had a normal histological structure (Figure 1A). In contrast, the colon sections from the group with induced colon cancer contained tumor cells indicative of anaplasia, dysplasia, and hyperchromasia in the lumen (Figure 1B). The rats with induced colon cancer that were treated with MSCs exhibited a noticeable enhancement in the lining epithelium of the mucosa, glandular structure, and a few inflammatory cells (Figure 1C). On the other hand, the rats with induced colon cancer that were treated with MSCs displayed a healthy structure (Figure 1D).

### MSC treatment improves the histology in rats with colon cancer

**Figure 2:**
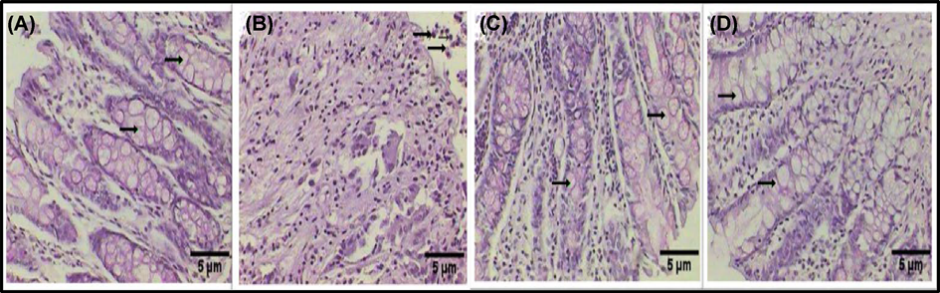
Histological examination of the colon section stained with PAS Examination of the histological changes of the colon section of a control rat exhibiting a normal intestinal glandular structure with normal aberrance of goblet cells (black arrows) (**A**). A photomicrograph of a rat colon after tumor induction by DMH (20 mg/kg), exhibiting malformation of the intestinal glandular structure, absence of goblet cells, and tumor cells in the lumen (black arrows) (**B**). A photomicrograph of a rat colon after tumor induction by DMH (20 mg/kg), exhibiting a healthy structure with goblet cell (black arrows) and scattered inflammatory cells (**C**). A photomicrograph of a rat colon exhibiting a healthy structure with goblet cell (black arrows) (**D**) (PAS: 400×).

In the control group, the colon sections that were stained with PAS displayed normal intestinal glandular structures with normal aberrance of goblet cells (Figure 2A). In contrast, the malformation of the intestinal glandular structure, absence of goblet cells, and tumor cells in the lumen were observed in the rats with induced colon cancer (Figure 2B). A significant improvement was observed in the PAS-stained colon sections of the rats with induced colon cancer that were treated with MSCs (Figure 2C and D).

**Figure 3:**
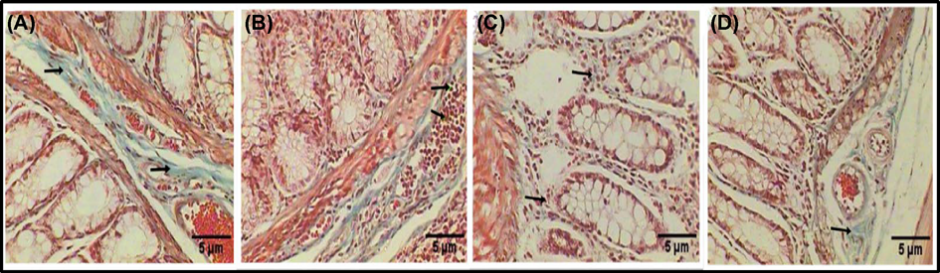
Histological examination of the colon section stained with Masson’s trichrome Examination of the histological changes of the colon section of a control rat exhibiting a normal structure with collagenous fiber content in the periglandular cells in the mucosal layer and lamina propria (black arrows) (**A**). A photomicrograph of a rat colon after tumor induction by DMH (20 mg/kg), exhibiting aggregations of leukocytic inflammatory cells mixed with tumor cells (black arrows) (**B**). A photomicrograph of a rat colon after tumor induction by DMH (20 mg/kg), exhibiting scattered inflammatory cells mixed with tumor cells and a clear enhancement in the collagenous contented in all layers (**C**). A photomicrograph of a rat colon exhibiting a healthy structure and moderate increase in collagenous content in all layers (**D**) (Masson’s trichrome: 400×).

Staining with Masson’s trichrome revealed that the colon sections of the control group exhibited a normal structure with collagenous fiber content in the periglandular cells in the mucosal layer and lamina propria (Figure 3A). In contrast, the colon section from the rats with induced colon cancer exhibited the aggregation of leukocytic inflammatory cells with tumor cells (Figure 3B). Meanwhile, the group with induced colon cancer that was treated with MSCs exhibited scattered inflammatory cells mixed with tumor cells and a noticeable improvement in the collagenous content in all the layers (Figure 3C). Lastly, a healthy structure and moderate increase in collagenous content in all the layers were observed in the group treated with MSC (Figure 3D).

### Effect of MSCs on the level of cytokeratin in rats with colon cancer

**Figure 4:**
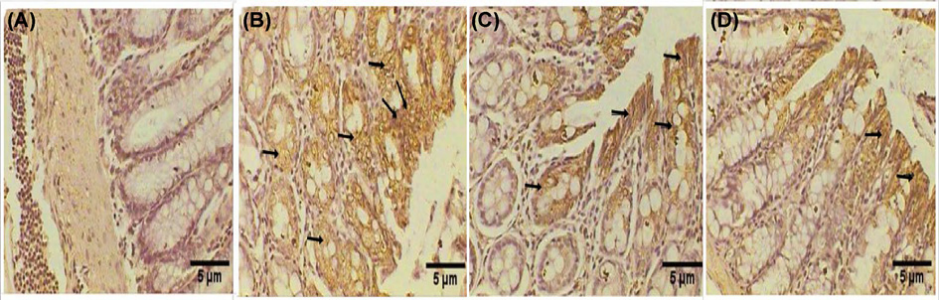
Immunohistochemical examination of the colon section Immunohistochemical examination of the colon section of a control rat exhibiting no response to anti-cytokeratin 20 in the large intestine of rat (**A**). Rat colon after tumor induction by DMH (20 mg/kg), exhibiting intense immune response to anti-Cytokeratin 20 (black arrows) (**B**). A photomicrograph of the colon section of colon cancer-prompted rat treated with DMH, exhibiting a moderate immune response to anti-cytokeratin 20 (black arrows) (**C**). A photomicrograph of colon treated rat exhibiting a weak response to anti-cytokeratin 20 (black arrows) (**D**) (400×).

Since abnormal cytokeratin expression is observed in neoplasia and *Krt20* expression is observed in colorectal cancer, the colon sections underwent immunohistochemical examination for cytokeratin 20. The colon sections from the control group were not stained by the anti-CK20 antibody (Figure 4A). In contrast, the colon sections from the group with induced colon cancer had intense staining of the anti-cytokeratin 20 body (Figure 4B). In the group with induced colon cancer that was treated with MSCs, a moderate positive reaction to anti-cytokeratin 20 in the glandular cells of the mucosal layer was observed (Figure 4C). On the other hand, there was a weak response to the anti-cytokeratin 20 antibody in the group treated with MSCs alone (Figure 4D).

### Effect of MSCs on the level of oxidative stress in rats with colon cancer

**Figure 5:**
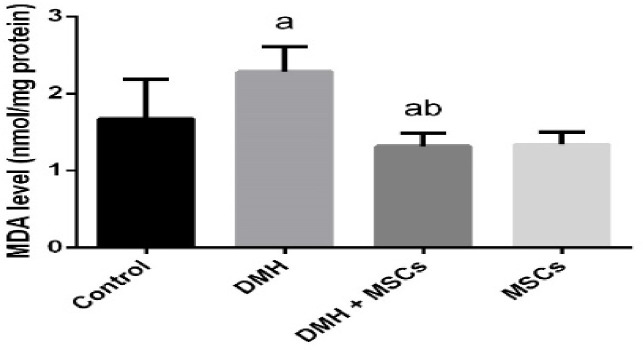
Oxidative stress markers Malondialdehyde (MDA) in the rat colon treated with MSC and DMH-prompted colon cancer. Values are expressed as mean ± standard deviation (SD) (*n*=10). ^a^Significant variation at *P*<0.05 represented with respect to the control group. ^b^Significant variation at *P*<0.05 represented with respect to the DMH group.

**Figure 6:**
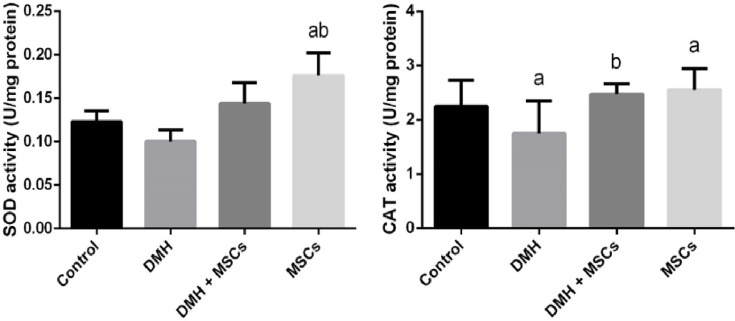
Enzymatic antioxidant status Antioxidant enzyme activities in the rat colon treated with MSC and DMH-prompted colon cancer. Values are expressed as mean ± SD (*n*=10). ^a^Significant change at *P*<0.05 represented with respect to the control group. ^b^Significant change at *P*<0.05 represented with respect to the DMH group.

Since oxidative stress is associated with colon cancer, the effect of MSCs on colon cancer was examined by comparing the oxidative stress levels in the rats in the different groups (Figure 5). The higher level of LPO in the colon homogenates of the rats treated with DMH indicated an increase in oxidative stress compared with the control group. But such an increase in the level of LPO was inhibited in the groups treated with DMH and MSC or only MSC. Furthermore, the administration of DMH was revealed to inhibit the activities of SOD and CAT (*P*<0.05). Interestingly, treatment with MSC significantly increased the activities of SOD and CAT (Figure 6).

### Effect of MSCs on the level of apoptosis in rats with colon cancer

**Figure 7:**
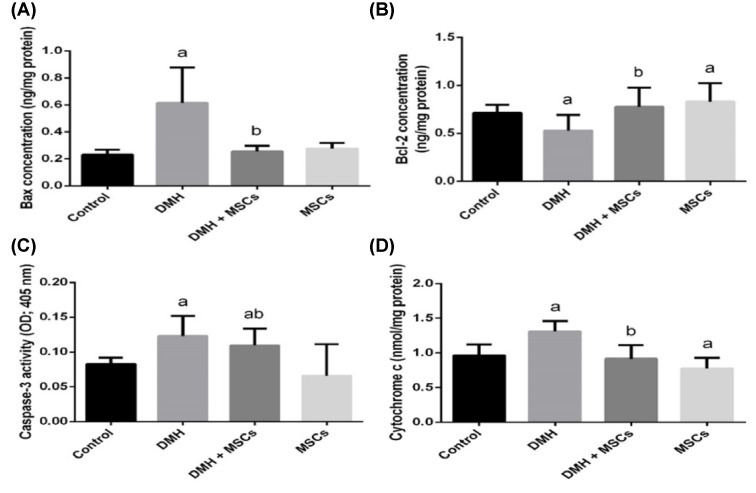
Colorectal apoptotic biomarkers Antiapoptotic/proapoptotic protein level markers (**A,B**) in rat colon treated with MSC and DMH-induced colon cancer. Caspase-3 and cytochrome-*c* activities in rat colon treated with MSC and DMH-induced colon cancer (**C,D**). Values are expressed as mean ± SD (*n*=10). ^a^Significant variation at *P*<0.05 represented with respect to the control group. ^b^Significant variation at *P*<0.05 represented with respect to the DMH group.

The antiapoptotic effects of MSC treatment in rats were examined by measuring the level of antiapoptotic proteins such as Bcl-2 and pro-apoptotic proteins such as Bax, caspase-3, and cytochrome-*c* in the colon homogenates (Figure 7). The level of Bax in the DMH group with induced colon cancer was higher than in the control group, whereas the level of Bcl-2 was significantly increased in the groups treated with DMH and MSC or only MSC (*P*<0.05). Notably, caspase-3 and cytochrome-*c* activities exhibited a pattern similar to Bax; they were significantly increased compared with those in the control group. However, such an increase in caspase-3 and cytochrome-*c* was inhibited by MSC in the groups treated with DMH and MSC or only MSC.

### Effect of MSCs on cell proliferation in rats with colon cancer

**Figure 8:**
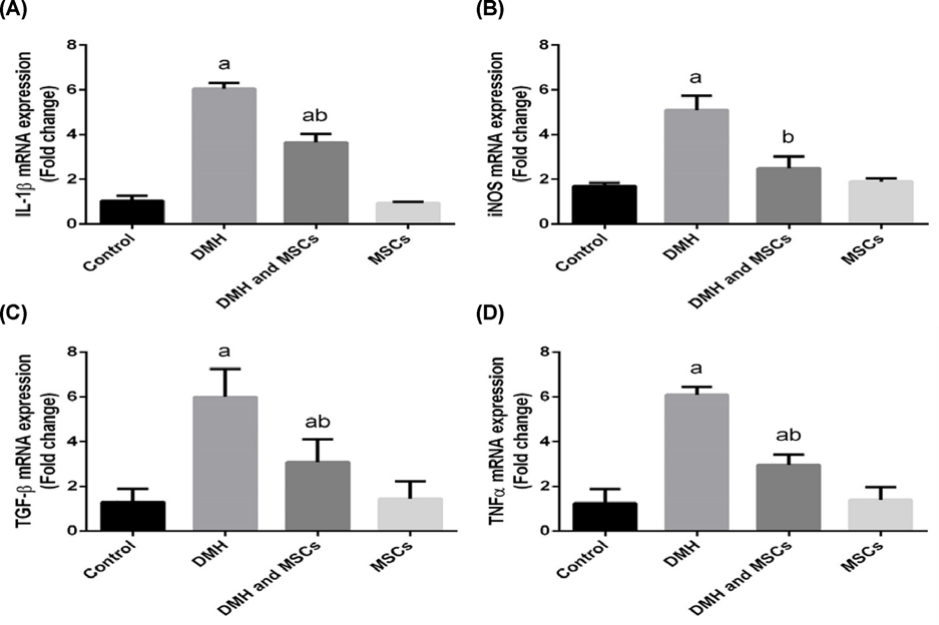
Gene expression analysis IL-1β mRNA, iNOS mRNA, TGF-β mRNA, and TNFα mRNA expressions in rat colon treated with MSC and DMH-prompted colon cancer. Values are expressed as mean ± SD (*n*=10). ^a^Significant variation at *P*<0.05 represented with respect to the control group. ^b^Significant variation at *P*<0.05 represented with respect to the DMH group. (**A**) IL-1β, (**B**) iNOS, (**C**) TGF-β, (**D**) TNFα.

The mechanisms underlying the inhibition of colorectal carcinogenesis by the MSCs were further dissected. We quantified the expressions of *Nos2, Ptgs2, Tnf, Tgfb, Il1b*, and *Il6*, encoding inducible nitric oxide synthase (iNOS), COX-2, tumor necrosis factor α (TNF-α), transforming growth factor β (TGF-β), interleukin (IL)-1β, and IL-6, respectively, in the colon tissues using RT-PCR. A significant up-regulation of *Il1b, Nos2, Tgfb*, and *Tnf* was observed in the group induced by DMH compared with the control group. Contrary to our data, treatment with MSC has been reported to down-regulate the genes in the groups treated with DMH and MSC or only MSC (Figure 8).

## Discussion

CRC is the second most common cause of cancer-related deaths worldwide. The present study investigated whether BMCs could reduce the risk of DMH-induced colon cancer by examining the effect of MSCs on the level of oxidative stress, inflammation and apoptosis, as well as the epithelial structures in the rats with colon cancer.

Oxidative stress is associated with colon cancer; ROS production is known to cause an oxidant/antioxidant imbalance. In the present study, the response of the primary oxidative stress markers was found to increase during LPO, consistent with previous data [26,27]. The enhancement of LPO was associated with the decreased levels of antioxidant enzymes, namely, SOD and CAT. The antioxidant enzymes have been reported to constitute a mutually supportive defense system against ROS. Here, we demonstrated that DMH induced a significant decrease in the activities of antioxidant enzymes, accumulating superoxide radicals and further stimulating LPO. The inhibition of antioxidant enzymes by DMH was likely caused by protein inactivation by the ROS, as oxidative damage often leads to loss of specific protein functions [28]. CRC reduces the antioxidant capacity of the rat colon, as evidenced by the decreased activity of the antioxidant enzymes. Our data are in agreement with other studies [29].

Inflammation plays a significant role in the initiation and promotion of cancer and affects other related processes, including apoptosis, angiogenesis, cancer cell invasion, cell cycle promotion, and metastasis [30]. Here we observed a significant up-regulation of *Nos2*, encoding the pro-inflammatory enzyme iNOS, in the rats induced by DMH. Our findings are consistent with the observation that DMH-induced CRC exhibits a high level of expression of *Nos2* [31] and the numerous reports of high iNOS activity in colon cancer [32,33].

We also observed a significant up-regulation of pro-inflammatory genes encoding iNOS, COX-2, TNF-α, TGF-β, IL-1β, and IL-6 in the group induced by DMH. These genes are activated by nuclear factor-κB (NF-κB), which has a significant role in immunity. NF-κB is triggered by ubiquitination, phosphorylation, and the subsequent proteolytic squalor of the IκB by the activated IκB kinase (IKK) [34]. The liberated NF-κB translocates to the nucleus and binds to the κB motifs as a transcription factor in the promoters of target genes, leading to their transcription.

Aberrant NF-κB activity is associated with various inflammatory diseases. Most anti-inflammatory drugs suppress the production of inflammatory cytokines by inhibiting the NF-κB pathway [35]. The results presented in this study revealed that the *in vivo* induction with DMH resulted in a robust increase in pro-inflammatory cytokine levels and the expression of the genes encoding iNOS, TNF-α, TGF-β, and IL-1β. However, treatment with MSCs was found to prevent inflammation, likely due to the capability of MSCs to prevent NF-κB’s translocation to the nucleus [36–38].

CRC development is characterized by a sequence of events during which the normal colonic epithelium is gradually transformed into invasive carcinoma tissues, usually via the development of colorectal adenomas. This sequence of events is triggered by the accumulation of molecular epigenetic and genetic alterations that cause progressive disorders in cell growth, differentiation, and apoptosis [39,40]*.* Abnormalities in apoptotic function have been identified as critical events in the pathogenesis of CRC and its resistance to chemotherapeutic drugs and radiotherapy [41,42].

Here, DMH-induced apoptosis and Bax, Bcl-2, and caspase-3 protein levels were measured in the colon homogenates. ROS increases the permeability of mitochondrial membranes, resulting in mitochondrial failure [43]. The mitochondrial membrane’s permeability depends on the mitochondrial permeability transition pores that release cytochrome-*c* from the mitochondria to the cytosol [44]. Once released, cytochrome-*c* binds to Apaf-1 in the cytoplasm, forming a complex that activates caspase-9 with the subsequent activation of the apoptosis-inducing caspase-3 [45]. In our study, we observed DMH-induced apoptosis through the activation of caspase-3 in rat colons. Moreover, treatment with MSCs decreased caspase-3 activity. These results indicate that MSCs exert antiapoptotic effects, at least in part, by inhibiting caspase-3 activation.

On the other hand, Bcl-2 may counteract damage by reducing the LPO triggered by cytotoxic stimuli like ROS [46]. Bcl-2 is also found to prevent cytochrome-*c* release. Contrarily, Bax regulates apoptosis, not only through dimerization with the antiapoptotic Bcl-2 proteins but also through the regulation of cytochrome-*c* release and the subsequent caspase-3 activation [47]. Our results revealed that treatment with MSC reversed the altered levels of Bcl-2 and Bax induced by DMH and substantially restored the Bcl-2/Bax ratio. In the present study, the treatment with MSCs inhibited all toxic events induced by DMH. MSC also scavenges oxygen and nitrogen-based reactant generated in the mitochondria, stabilizes the mitochondrial membrane, and enhances antiapoptotic signaling.

The treatment of MSCs also helps restore the structure of the epithelium in colon cancer. CK20 is a member of the cytokeratin family that includes at least 20 types of cytoplasmic intermediate filaments. The abnormal expression of cytokeratin genes has been observed in neoplasia and other diseases [48]. In addition, *Krt20* expression has been observed in most colorectal and gastric cancers [49–52]. In the present study, we observed strong CK20 immunostaining in the colorectal tissues of rats treated with DMH compared to the untreated ones.

On the other hand, we observed a complete regeneration of the mucosal epithelium and a significant improvement in collagen fiber accumulation in the arts with induced colon cancer and treated with MSCs. Moreover, mucus secretion in the goblet cells was detected in the DMH-induced rats following treatment with MSC. Thus, MSCs treatment may potentiate the repair of the mucosal epithelial cells. MSCs have been found to collect in inflamed areas of the colon and contribute to tissue repair by differentiating into endothelial cells, vascular smooth muscle cells, pericytes, or epithelial cells [53]; this finding agrees with ours.

MSCs treatment may also be useful in antifibrotic therapies, where it can prevent fibrosis by producing basic fibroblast growth factor (bFGF) [53]. Many studies have confirmed that MSCs possess unique immunologic characteristics. The transplantation of allogeneically incompatible MSCs to adult animals has enabled engraftment in murine models [54–57]. Also, the rats engrafted with rat MSCs showed excellent survival. MSC also promotes tissue renewal and repair through the synergistic down-regulation of pro-inflammatory cytokines, inhibition of epithelial cell apoptosis, suppression of tumor growth, and enhancement of antioxidant properties.

Moreover, it has been recommended that engrafted MSCs also differentiate into, secrete vascular endothelial growth factor, colonic interstitial lineage cells and TGF-β1, which both play a significant role in the curative of injured colonic mucosa [56]. Also, the MSCs allografted to the sites of Kaposi’s sarcoma substantially prevent tumor growth by down-regulating protein kinase B (AKT) activity [58,59]. Furthermore, cancer cells may express proteins that can trigger signaling pathways that attract MSCs and facilitate their journey to the tumor site [60].

## Conclusion

Our study reveals that MSCs exert tumor-suppressive effects and decrease the risk of DMH-induced colorectal carcinogenesis in rats, as shown by a decrease in the augmented release of inflammatory mediators, the increase in antioxidant mechanisms, and the enhancement of the immune response. These results suggest that MSCs may be a novel therapeutic intervention against DMH-induced colon cancer.

## Data Availability

The data used to support the findings of the present study are included within the article.

## Abbreviations

BAX: BCL2 associated X protein
BCL2: B-cell lymphoma protein 2
BMC: Bone marrow cell
BM-MSC: bone marrow-mesenchymal stem cell
COX: Cyclooxygenase
CRC: Colorectal cancer
DMH: 1,2-dimethylhydrazine
ELISA: enzyme-linked immunosorbent assay
HSC: hematopoietic stem cell
IL: interleukin
IκB: IκB inhibitor of nuclear factor kappa B
iNOS: inducible nitric oxide synthase
LPO: lipid peroxidation
MSC: mesenchymal stem cell
α-MEM: Alpha Minimum Essential Medium
NF-κB: nuclear factor-κB
PAS: Periodic acid–Schiff
PSN: Penicillin-Streptomycin-Neomycin
RT-PCR: real-time polymerase chain reaction
ROS: reactive oxygen species
SOD: superoxide dismutase
TGF-β: transforming growth factor β
TNF-α: tumor necrosis factor α
